# Supplementary material for: HIVprotI: an integrated web based platform for prediction and design of HIV proteins inhibitors
Source: J Cheminform. 2018 Mar 9;10:12. doi: 10.1186/s13321-018-0266-y (PMC5845081; doi:10.1186/s13321-018-0266-y)
Supplement: Supplementary file 2 — Additional file 2. Source code of HIVProtI web server. [file 13321_2018_266_MOESM2_ESM.zip › HIVprotI_Source-code/hivprotI/weka-3-6-6/doc/weka/attributeSelection/ConsistencySubsetEval.hashKey.html]

ConsistencySubsetEval.hashKey


---


|  |  |  |
| --- | --- | --- |
| **Overview** \| **Package** \| **Class** \| **Tree** \| **Deprecated** \| **Index** \| **Help** \| **Weka's home** | |  |
| **PREV CLASS**   **NEXT CLASS** | **FRAMES**    **NO FRAMES**     **All Classes** |  |
| SUMMARY: NESTED \| FIELD \| CONSTR \| METHOD | DETAIL: FIELD \| CONSTR \| METHOD |  |


---


## weka.attributeSelection Class ConsistencySubsetEval.hashKey

```
java.lang.Object
  weka.attributeSelection.ConsistencySubsetEval.hashKey
```

**All Implemented Interfaces:**: java.io.Serializable, RevisionHandler

**Enclosing class:**: ConsistencySubsetEval

---

``` public class ConsistencySubsetEval.hashKey extends java.lang.Object implements java.io.Serializable, RevisionHandler ```

Class providing keys to the hash table.

**See Also:**: Serialized Form

---

**Constructor Summary**

`ConsistencySubsetEval.hashKey(double[] t)`
  
          Constructor for a hashKey

`ConsistencySubsetEval.hashKey(Instance t,
int numAtts)`
  
          Constructor for a hashKey


| **Method Summary** | |
| --- | --- |
| `boolean` | `equals(java.lang.Object b)`             Tests if two instances are equal |
| `java.lang.String` | `getRevision()`             Returns the revision string. |
| `int` | `hashCode()`             Calculates a hash code |
| `void` | `print_hash_code()`             Prints the hash code |
| `java.lang.String` | `toString(Instances t, int maxColWidth)`             Convert a hash entry to a string |

**Methods inherited from class java.lang.Object**

`getClass, notify, notifyAll, toString, wait, wait, wait`

**Constructor Detail**

### ConsistencySubsetEval.hashKey

```
public ConsistencySubsetEval.hashKey(Instance t,
                                     int numAtts)
                              throws java.lang.Exception
```

:   Constructor for a hashKey

    **Parameters:**: `t` - an instance from which to generate a key: `numAtts` - the number of attributes **Throws:**: `java.lang.Exception` - if something goes wrong

---


### ConsistencySubsetEval.hashKey

```
public ConsistencySubsetEval.hashKey(double[] t)
```

:   Constructor for a hashKey

    **Parameters:**: `t` - an array of feature values


**Method Detail**

### toString

```
public java.lang.String toString(Instances t,
                                 int maxColWidth)
```

:   Convert a hash entry to a string

    :   **Parameters:**: `t` - the set of instances: `maxColWidth` - width to make the fields **Returns:**: the hash entry as string

---


### hashCode

```
public int hashCode()
```

:   Calculates a hash code

    :   **Overrides:**: `hashCode` in class `java.lang.Object`
    :   **Returns:**: the hash code as an integer

---


### equals

```
public boolean equals(java.lang.Object b)
```

:   Tests if two instances are equal

    :   **Overrides:**: `equals` in class `java.lang.Object`
    :   **Parameters:**: `b` - a key to compare with **Returns:**: true if the objects are equal

---


### print\_hash\_code

```
public void print_hash_code()
```

:   Prints the hash code

---


### getRevision

```
public java.lang.String getRevision()
```

:   Returns the revision string.

    :   **Specified by:**: `getRevision` in interface `RevisionHandler`
    :   **Returns:**: the revision


---


|  |  |  |
| --- | --- | --- |
| **Overview** \| **Package** \| **Class** \| **Tree** \| **Deprecated** \| **Index** \| **Help** \| **Weka's home** | |  |
| **PREV CLASS**   **NEXT CLASS** | **FRAMES**    **NO FRAMES**     **All Classes** |  |
| SUMMARY: NESTED \| FIELD \| CONSTR \| METHOD | DETAIL: FIELD \| CONSTR \| METHOD |  |


---
